# *Notes from the Field:* Cruise Ship Norovirus Outbreak Associated with Person-to-Person Transmission — United States Jurisdiction, January 2023

**DOI:** 10.15585/mmwr.mm7230a5

**Published:** 2023-07-28

**Authors:** Carolyn A. Crisp, Keisha A. Jenkins, Ian Dunn, Andrew Kupper, Jona Johnson, Stefanie White, Erin D. Moritz, Luis O. Rodriguez

**Affiliations:** ^1^Epidemic Intelligence Service, CDC; ^2^Division of Environmental Health Science and Practice, National Center for Environmental Health, CDC; ^3^Geospatial Research, Analysis, and Services Program, Agency for Toxic Substances and Disease Registry, Atlanta, Georgia.

CDC’s Vessel Sanitation Program (VSP) monitors cases of acute gastroenteritis (AGE) on board cruise ships traveling to a U.S. port (*1*). Persons who have ≥3 loose stools (or more than normal for that person) within a 24-hour period or vomiting plus one other sign or symptom (e.g., fever, diarrhea, bloody stool, myalgia, abdominal cramps, or headache) meet the case definition for reportable AGE (*2*). When the percentage of passengers or crew members with AGE is ≥2% and the ship is due to arrive at a U.S. port within 15 days, the Maritime Illness Disease Reporting System alerts VSP and activates an investigation (*1*). During the first week of January 2023, VSP was notified of cases of AGE affecting >2% of passengers on board a ship that had completed three voyages in Europe and was within 15 days of arriving at a U.S. port (voyage 4)* (Figure). Ship medical crew members submitted stool samples from ill travelers for testing. All samples tested positive for norovirus genotype II. While the ship was sailing to a U.S. port, VSP monitored AGE cases on board and reviewed case data. By mid-January, passenger AGE prevalence reached 3.4%.

**FIGURE:**
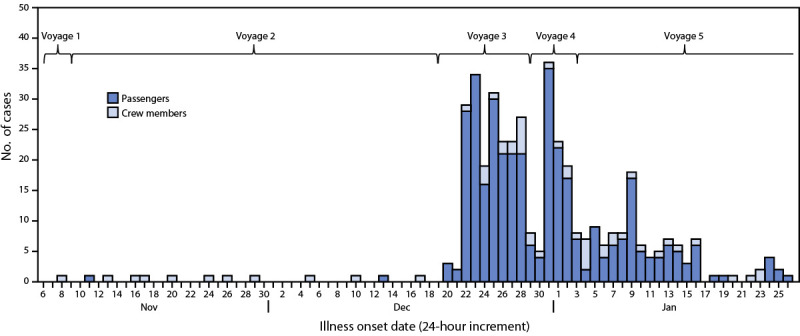
Cases of acute gastroenteritis (N = 410),* by illness onset date^†^ — Cruise ship A, five voyages, November 2022–January 2023^§^ * Cases occurred among 356 passengers and 54 crew members. ^†^ Index case likely occurred on November 8, 2022. ^§^ Voyage 5 was a world voyage that lasted ≥30 days.

## Investigation and Outcomes

During mid-January 2023, VSP’s outbreak team boarded the ship to conduct an epidemiological and environmental investigation. The investigation focused on exposure sources and routes of transmission. Occupational and social behaviors of crew members were evaluated because the epidemic curve (Figure) suggested that the index case occurred in a crew member during voyage 1 who developed symptoms after embarking, likely leading to transmission among other crew members (voyage 2), and then to passengers (voyage 3). After the investigation, VSP continued to monitor the ship (voyage 5) until it left U.S. jurisdiction. This activity was reviewed by CDC and was conducted consistent with applicable federal law and CDC policy.^†^

Among 410 reported cases during November 2022–January 2023 (voyages 1–5), 356 (87%) occurred in passengers and 54 (13%) in crew members. The index case likely occurred in a food and beverage crew member sailing on a crew-only voyage (voyage 1). In general, crew members with AGE reported to onboard medical personnel in a timely manner and were isolated until 48 hours after symptoms subsided. Crew member transmission was followed by passenger transmission on voyages 3, 4, and 5. Vomiting and diarrhea were the predominant symptoms among cases. Approximately 70% of crew members with AGE interacted with passengers (i.e., housekeeping and food and beverage services). VSP partnered with the United States Agency for Toxic Substances and Disease Registry’s Geospatial Research, Analysis, and Services Program to create four-dimensional visual models of the ship. These models helped visualize continued norovirus transmission and sources of potential exposure (e.g., contaminated surfaces in cabins of persons with AGE and high-touch surfaces in common areas).

## Preliminary Conclusions and Actions

During this investigation, VSP used surveillance data and environmental and spatial analyses to improve field responses and quickly identify sources of norovirus exposure and transmission. Public health response to maritime AGE outbreaks involves robust and timely monitoring of AGE cases and collaborations with cruise companies. To prevent illness transmission across voyages, cruise ship personnel and travelers should always maintain proper hand hygiene and sanitation practices, and passengers and crew members should immediately isolate and report illness symptoms to the ship medical center (*3*). Cruise companies are encouraged to conduct frequent norovirus trainings for crew members, especially those with limited experience working with the cruise company (e.g., those who have served fewer than three contract terms).

## Footnotes

* Voyage 1 (early November 2022), voyage 2 (early November–mid-December 2022), voyage 3 (mid-to-late December 2022), voyage 4 (late December 2022–early January 2023) and voyage 5 (began in early January and lasted ≥30 days); the ship made port in the United States during mid-January 2023.

† 45 C.F.R. part 46, 21 C.F.R. part 56; 42 U.S.C. Sect. 241(d); 5 U.S.C. Sect. 552a; 44 U.S.C. Sect. 3501 et seq.

## References

[1] Jenkins KA, Vaughan GH Jr, Rodriguez LO, Freeland A. Acute gastroenteritis on cruise ships—Maritime Illness Database and Reporting System, United States, 2006–2019. MMWR Surveill Summ 2021;70(No. SS-6):1–19. 10.15585/mmwr.ss7006a1 34555008 PMC8480991

[2] Freeland AL, Vaughan GH Jr, Banerjee SN. Acute gastroenteritis on cruise ships—United States, 2008–2014. MMWR Morb Mortal Wkly Rep 2016;65:1–5. 10.15585/mmwr.mm6501a1 26766396

[3] CDC. Vessel sanitation program: illness prevention resources. Atlanta, GA: US Department of Health and Human Services, CDC; 2022. https://www.cdc.gov/nceh/vsp/healthy.htm

